# BLX‐1002 restores glucose sensitivity and enhances insulin secretion stimulated by GLP‐1 and sulfonylurea in type 2 diabetic pancreatic islets

**DOI:** 10.14814/phy2.12014

**Published:** 2014-05-28

**Authors:** Qimin Zhang, Fan Zhang, Åke Sjöholm

**Affiliations:** 1 Department of Internal Medicine, Södertälje Hospital, Södertälje, Sweden; 2 Department of Biochemistry and Molecular Biology, College of Medicine, University of South Alabama, Mobile, Alabama

**Keywords:** Diabetes, islet, PPAR, PI3K

## Abstract

BLX‐1002 is a novel thiazolidinedione with no peroxisome proliferator‐activated receptor (PPAR) activity that has been shown to improve glycemia in type 2 diabetes without weight gain. We previously found that BLX‐1002 selectively augments glucose‐sensitive (but not basal) insulin secretion in normal mouse *β*‐cells. We have now extended these observations to other insulin secretagogues and to diabetic rat islets. To this end, dynamics of insulin secretion stimulated by glucose, GLP‐1, and the sulfonylurea tolbutamide were examined in pancreatic islets from nondiabetic Wistar and type 2 diabetic Goto‐Kakizaki rats ex vivo. BLX‐1002 restored normal glucose‐sensitive insulin secretion in otherwise “glucose‐blind” islets from GK rats, but did not affect basal or glucose‐stimulated secretion in normal Wistar rat islets. The stimulatory effect of BLX‐1002 on insulin secretion at high glucose required Ca^2+^ and involved phosphatidylinositol 3‐kinase (PI3K) activity. Consistent with its effects on insulin secretion, BLX‐1002 also augmented insulin secretion and cytoplasmic‐free Ca^2+^ concentrations ([Ca^2+^]_i_) stimulated by high glucose, GLP‐1, and tolbutamide in islets from GK, but not Wistar, rats. The inactive analog BLX‐1237 had no effects. In conclusion, our findings suggest that BLX‐1002 potentiates insulin secretion by different stimuli in diabetic *β*‐cells only, in a Ca^2+^‐dependent manner and involving PI3K.

## Abstract (precis)

The non‐PPAR TZD agent BLX‐1002 restores normal glucose‐sensitive insulin secretion in otherwise “glucose‐blind” islets from diabetic Goto‐Kakizaki rats, but does not affect basal or glucose‐stimulated secretion in normal Wistar rat islets. BLX‐1002 also augments insulin secretion stimulated by GLP‐1 and tolbutamide in islets from GK, but not Wistar, rats.

## Introduction

Diabetes mellitus is a syndrome characterized by disordered metabolism resulting in hyperglycemia. Type 2 diabetes (T2DM) is by far the most common form of the disease, resulting from a combination of impaired insulin production and insulin resistance in peripheral tissues (Zimmet et al. 2001). Diabetes is widespread and it is the fourth leading cause of death in the United States. According to the World Health Organization and International Diabetes Federation, its incidence is increasing rapidly and is estimated to reach 560 million people by the year 2030 (www.idf.org). The expenses to diabetes have been shown to be a major drain on health‐ and productivity‐related resources for healthcare systems and governments. In the United States alone, the annual cost for diabetes amounts to $245 billion, of which ~97% is targeted to T2DM (American Diabetes Association 2013).

While insulin resistance is a cardinal pathogenic feature of T2DM, affecting 90–95% of patients, diabetes does not occur until the pancreatic islet *β*‐cell no longer produces insulin in a rate sufficient to fully compensate for the impaired insulin sensitivity (Weir 1995). Unfortunately, T2DM is a progressive disease characterized by a gradual loss of *β*‐cell function. It is estimated that, at the time of T2DM diagnosis, 50% of insulin production is already lost (U.K. prospective diabetes study 16 1995). Therefore, finding ways of halting disease progression by improvement or preservation of *β*‐cell function is a major goal in contemporary diabetes research and in the clinical management of T2DM.

The *β*‐cells show several defects in T2DM. Early in the course is loss of first phase glucose‐stimulated insulin secretion (GSIS), required to reduce hepatic glucose production, thereby controlling prandial glycemia (Pfeifer et al. 1981). Remarkably, this loss of early insulin secretion appears restricted to glucose; in effect, *β*‐cells become selectively “blind” to glucose (Malaisse 1994). Later phases of insulin secretion, however, are reduced not only in response to glucose, but also other secretagogues in T2DM (Porte and Kahn 2001).

The lean Goto‐Kakizaki (GK) rat is a well‐established model for nonobese human T2DM (Portha et al. 2009). It is characterized primarily by loss of *β*‐cell function, more specifically impaired GSIS (Ghanaat‐Pour et al. 2007), whereas insulin resistance is less a prominent feature in this model.

BLX‐1002 is a novel, antidiabetic, water‐soluble thiazolidinedione with no structural resemblance with any existing compounds (Zhang et al. 2009). Its structure is described in U.S. Patent # 6794401. It does not affect peroxisome proliferator–activated receptors (PPAR) (Zhang et al. 2009). BLX‐1002 improves hyperglycemia in diabetic animal models without body weight gain as otherwise commonly noted in patients on PPAR agonist treatment (Zhang et al. 2009).

We previously found that BLX‐1002 selectively augments glucose‐sensitive (but not basal) insulin secretion from normal mouse *β*‐cells, by mechanisms involving PI3K (Zhang et al. 2009). We have now extended these observations to other insulin secretagogues and to diabetic islets. To this end, we aimed to investigate the dynamics of insulin secretion stimulated by physiologically and therapeutically important stimuli (viz. glucose, GLP‐1, and the sulfonylurea tolbutamide) in pancreatic islets from nondiabetic Wistar and type 2 diabetic Goto‐Kakizaki rats in vitro.

## Materials and Methods

### Materials

BLX‐1002 and BLX‐1237 were graciously donated by Bexel Pharmaceuticals, Inc. (Union City, CA). LY‐294002 was purchased from Calbiochem (La Jolla, CA). Wortmannin, acetylcholine chloride, Fura‐2/acetoxymethylester (Fura‐2/AM) and EGTA were from Sigma‐Aldrich (St. Louis, MO). GLP‐1 (7–36) was from PolyPeptide Laboratories (Torrance, CA). Collagenase A was from Roche Diagnostics (Mannheim, Germany) and Bio‐Gel P‐4 (fine, 65 ± 20 *μ*m, wet) was from Bio‐Rad Laboratories (Hercules, CA). Rat insulin ELISA kits were from Mercodia (Uppsala, Sweden). RPMI‐1640 culture medium and FBS were from Life Technologies Invitrogen (Paisley, UK).

### Preparation of pancreatic islets

Goto‐Kakizaki (GK) rats, a nonobese model of type 2 diabetes, and normal Wistar rats were used in the present study. The GK rat is a Wistar substrain, which spontaneously develops type 2 diabetes in early life (Ghanaat‐Pour et al. 2007; Portha et al. 2009). Pancreatic islets were prepared from diabetic GK and control Wistar rats, ~3‐month‐old, purchased from Taconic Europe (barrier EBU 202, Bomholt site, Ry, Denmark). At this age, GK rats are mildly hyperglycemic and slightly insulinopenic (Ghanaat‐Pour et al. 2007; Hussain et al. 2014). Additionally, their GSIS is virtually absent (Ghanaat‐Pour et al. 2007). Islets were isolated by collagenase digestion (Ghanaat‐Pour et al. 2007) and cultured overnight. For measurement of cytosolic‐free Ca^2+^ concentration ([Ca^2+^]_i_), islets were dispersed into single cells by shaking in medium containing 1 mmol/L EGTA (Zhang et al. 2006a). Islets or cells were maintained overnight in RPMI‐1640 tissue culture medium in the presence of 5.5 mmol/L glucose, supplemented with 10% (v/v) FBS, 2 mmol/L l‐glutamine, 100 IU/mL penicillin, and 100 *μ*g/mL streptomycin. All experiments were conducted according to the “Guide for the Care and Use of Laboratory Animals” published by U.S. National Institutes of Health (NIH publication # 85–23, revised 1985) and approved by the regional ethics committee for animal experimentation (# S150‐04, S151‐04).

### Insulin secretion

#### Batch incubation

Islets were washed three times in KRBH (Krebs–Ringer bicarbonate Hepes) buffer containing (in mmol/L): 135 NaCl, 3.6 KCl, 5 NaHCO_3_, 0.5 NaH_2_PO_4_, 0.5 MgCl_2_, 1.5 CaCl_2_, and 10 Hepes, pH 7.4 with 0.1% BSA. Equal number of islets (Virkamäki et al. 1999) of comparable size was placed in each well of a 24‐well plate, preadded with 1 mL of the buffer in the presence of 10 *μ*mol/L BLX‐1002 or BLX‐1237 at 3 mmol/L glucose or 20 mmol/L glucose at 37°C. The plates with islets were incubated in an incubator for 30 min at 37°C. To investigate effects of kinase inhibitors on insulin secretion, islets were preincubated in the presence of the inhibitors (wortmannin [100 nmol/L] or LY‐294002 [10 *μ*mol/L]) or equal amount of vehicle (DMSO) at 3 mmol/L glucose for 15 min at 37°C, followed by addition of BLX‐1002 or BLX‐1037 and glucose to the desired concentration. The incubation was continued for 30 min. At the end of the incubation, plates were immediately transferred onto ice. The medium was collected and centrifuged and the supernatant was used for insulin assay by ELISA. The islets were collected and lysed for protein assay (Bio‐Rad).

#### Column perifusion

Dynamics of insulin secretion was studied by column perifusion as described (Zhang et al. 2006a). One hundred islets were carefully mixed with a small volume of prewetted Bio‐Gel P‐4, placed on the top of each parallel performed column, and perifused with KRBH buffer containing 0.1% BSA at a flow rate of 0.25 mL/min at 37°C. Islets were perifused with the buffer containing 3 mmol/L glucose, followed by addition of glucose and the agents to be tested. Fractions were collected every 2 min and the insulin content in each fraction was measured using rat insulin ELISA kits.

### Measurement of [Ca^2+^]_i_

[Ca^2+^]_i_ measurements were performed as previously described (Zhang et al. 2006a,b, 2009). Islet cells, prepared as above, were placed on glass coverslips and cultured overnight in RPMI medium. Before experiments, cells on coverslips were loaded with Fura‐2 (1.5 *μ*mol/L) for 20 min at 37°C in KRBH buffer in the presence of 3 mmol/L glucose and 0.1% BSA. The coverslips were subsequently rinsed once in the same buffer without the Ca^2+^ indicator and mounted at the bottom of a perifusion chamber on the stage of an inverted epifluorescence microscope (Olympus CK 40). The stage was thermostated to 37°C, and cells were superfused at a rate of 300 *μ*L/min with KRBH buffer containing 3 mmol/L glucose. Measurements of [Ca^2+^]_i_ were performed in cell clusters (3–4 cells) using a time‐sharing spectrofluorometer (RM‐5 system, PhotoMed) providing light flashes of 1‐msec duration at 340 and 380 nm every 10 msec. Fluorescence was recorded at 510 nm. While this setup, based on clusters of single cells, is well established (Zhang et al. 2006a,b, 2009), it cannot be excluded that loss of paracrine effects by non‐*β*‐cells due to disruption of normal islet architecture may influence the results.

### Statistics

Each assay was repeated a minimum of three times in duplicates. All data are presented as mean ± SEM. Statistical analyses of differences between groups were performed by ANOVA followed by the post hoc test Student–Newman–Keuls.

## Results

### BLX‐1002 restores glucose‐sensitive insulin secretion in islets from diabetic GK rats

**Figure 1.**
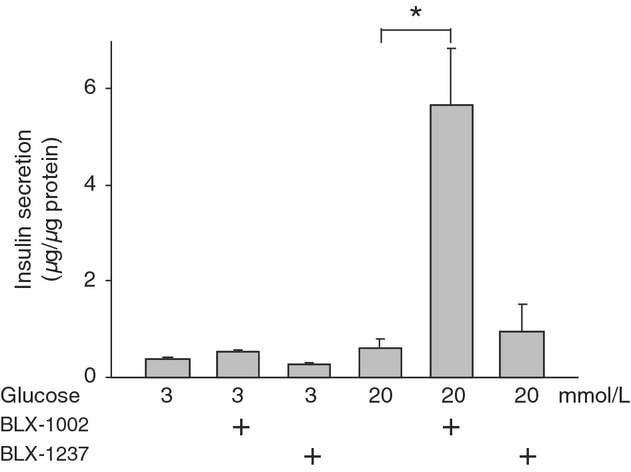
BLX‐1002, but not its structurally related compound, restores glucose‐sensitive insulin secretion in islets from diabetic GK rats. Pancreatic islets isolated from GK rats were incubated with various concentrations of glucose in the presence or absence of BLX‐1002 or BLX‐1237 (10 *μ*mol/L each) for 30 min. Insulin released to the medium was analyzed by ELISA. Bars represent mean ± SEM. Results are derived from three experiments (*P <**0.05 by ANOVA).

The effect of BLX compounds on insulin secretion was first studied in batch‐incubated pancreatic islets from the diabetic GK rats (Fig. 1). At low glucose, exposure of the islets to either BLX‐1002 or its structurally related compound BLX‐1237 did not alter insulin secretion. As expected (Ghanaat‐Pour et al. 2007), the diabetic islets did not respond to high glucose with increased insulin secretion. Coincubation of the islets with BLX‐1002 and high glucose for 30 min resulted in a dramatic enhancement of insulin secretion. At the concentration of 10 *μ*mol/L, BLX‐1002 induced insulin secretion approximately eightfold, compared to high glucose alone. In contrast, BLX‐1237 had no effect on insulin secretion at either low or high glucose.

### BLX‐1002 sensitizes the islets from diabetic GK rats to GLP‐1 and tolbutamide‐stimulated insulin secretion

**Figure 2.**
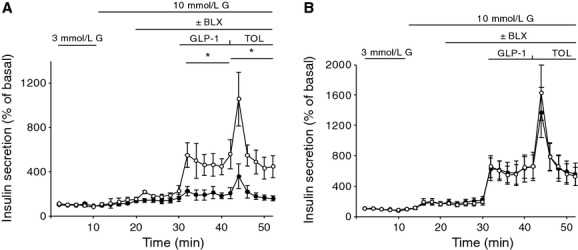
BLX‐1002 sensitizes islets from diabetic GK rats to GLP‐1‐ and tolbutamide‐stimulated insulin secretion. Equal numbers of islets isolated from diabetic GK (A) or healthy Wistar (B) rats were applied in microcolumns, preloaded with prewetted Bio‐gel P‐4. Islets were perifused with a buffer containing 3 mmol/L glucose, followed by additions of BLX‐1002 (10 *μ*mol/L), GLP‐1 (100 nmol/L), or tolbutamide ([Tol] 100 *μ*mol/L) as indicated. Fractions were collected every 2 min. Insulin content in each fraction was analyzed by ELISA. White dots represent BLX‐1002, black dots represent controls. Data represent mean ± SEM. Results are from four independent experiments. Based on mean values during stimulation period, *P <**0.05 versus controls (w/o BLX‐1002) by ANOVA.

Since GLP‐1‐based drugs and sulfonylurea compounds are important insulin secretagogues and widely used clinically in diabetes, we studied the effect of BLX‐1002 on GLP‐1‐ and tolbutamide‐induced insulin secretion (Fig. 2). The study was performed on islets prepacked in parallel performed microcolumns in order to reveal insulin secretion dynamics during stimulation by the agents. Perifusion of the islets from the diabetic GK rats with GLP‐1 or tolbutamide at 10 mmol/L glucose did not result in a significantly increased insulin secretion, as shown in Figure 2A. In the presence of BLX‐1002, however, the islets responded to the agents. At the concentrations of 100 nmol/L GLP‐1 and 100 *μ*mol/L tolbutamide, insulin secretion was increased threefold, compared to glucose alone (Fig. 2A). In contrast, while the islets from the nondiabetic Wistar rats responded normally to GLP‐1 and tolbutamide, addition of BLX‐1002 did not alter the secretory response to the agents (Fig. 2B).

### The effect of BLX‐1002 on glucose‐stimulated insulin secretion in islets from GK rats is Ca^2+^ dependent

**Figure 3.**
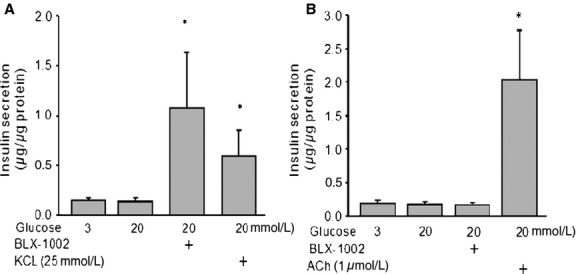
The effect of BLX‐1002 on glucose‐stimulated insulin secretion in islets from GK rats requires extracellular Ca^2+^. Islets isolated from GK rats were incubated in a buffer (A) with or (B) without Ca^2+^ with 2 mmol/L EGTA in the presence or absence of BLX‐1002 (10 *μ*mol/L), KCl, or acetylcholine (ACh) as indicated. Insulin secretion was analyzed by ELISA after 30 min incubation. Bars represent mean ± SEM. Results are derived from (A) six or (B) four experiments (*P <**0.05 by ANOVA).

In order to understand whether the effect of BLX‐1002 on glucose‐stimulated insulin secretion in the islets from diabetic rats is mediated through a Ca^2+^‐dependent mechanism, experiments were performed in the presence or absence of extracellular Ca^2+^. In the presence of extracellular Ca^2+^, BLX‐1002 – as expected – was able to induce insulin secretion when stimulatory concentration of glucose was added (Fig. 3A). When Ca^2+^ was depleted from the extracellular space, BLX‐1002 was no longer able to induce insulin secretion under these conditions, while the islets responded well to acetylcholine (Fig. 3B), which stimulates insulin secretion through Ca^2+^ mobilization from the intracellular Ca^2+^ stores (Selway et al. 2012). KCl (25 mmol/L), which directly depolarizes the cells causing Ca^2+^ influx, was included as a positive control as a non‐nutrient secretagogue.

### The effect of BLX‐1002 is associated with an increase in [Ca^2+^]_i_

**Figure 4.**
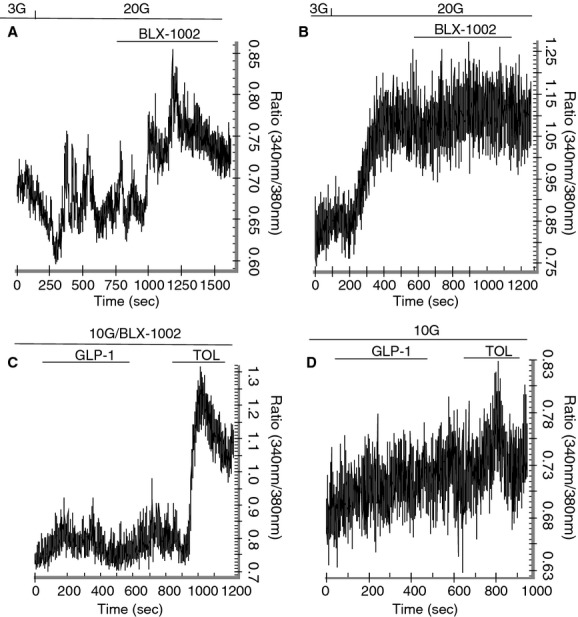
BLX‐1002‐induced insulin secretion is associated with an increase in [Ca^2+^]_i_. Experiments were performed as described in Materials and Methods. The upper panel shows islet cells isolated from (A) diabetic GK or (B) healthy Wistar rats perifused with a buffer containing 3 mmol/L glucose and stimulated with 20 mmol/L glucose, followed by BLX‐1002 (10 *μ*mol/L). Measurements of [Ca^2+^]_i_ were performed using a spectrofluorometer (RM‐5 system, PhotoMed), providing light flashes of 1‐msec duration at 340 and 380 nm every 10 msec. Fluorescence was recorded at 510 nm. Similar experiments were performed on islet cells from diabetic GK rats (C, D) perifused with a buffer containing 10 mmol/L glucose. Additions of GLP‐1 (100 nmol/L) or tolbutamide (100 *μ*mol/L) were made as indicated. Representative experiments of six for each are shown.

Since a rise in [Ca^2+^]_i_ is a crucial step in insulin secretion induced by glucose and many insulin secretagogues (Wollheim and Sharp 1981; Ashcroft and Rorsman 2012), [Ca^2+^]_i_ was measured during stimulation of the islet cells with glucose, tolbutamide, and GLP‐1 in the presence or absence of BLX‐1002 using the fluorescent Ca^2+^ probe Fura‐2. Consistent with the “blindness” to glucose in terms of insulin secretion (Fig. 1A), changes in [Ca^2+^]_i_ in islet cells from GK rats stimulated by the sugar was negligible (Fig. 4A). Addition of BLX‐1002 under these conditions resulted in a significant increase in [Ca^2+^]_i_ (Fig. 4A), again in good alignment with the restoration of GSIS conferred by BLX‐1002 (Fig. 1A). In contrast, islet cells from Wistar rats responded normally to glucose, showing a clear rise in [Ca^2+^]_i_ (Fig. 4B). Addition of BLX‐1002 did not change the [Ca^2+^]_i_ response (Fig. 4B). The effect of BLX‐1002 on GLP‐1‐ and tolbutamide‐induced rise in [Ca^2+^]_i_ was also studied under similar conditions. Islet cells from GK rats did not respond to GLP‐1 in terms of rise in [Ca^2+^]_i_ (Fig. 4D). Stimulation with tolbutamide elicited only a slight [Ca^2+^]_i_ increase (Fig. 4D). After treatment with BLX‐1002, however, [Ca^2+^]_i_ was enhanced by both GLP‐1 and tolbutamide (Fig. 4C), the latter evoking a robust rise in [Ca^2+^]_i_.

### The enhancing effect of BLX‐1002 on GSIS involves PI3K activity

**Figure 5.**
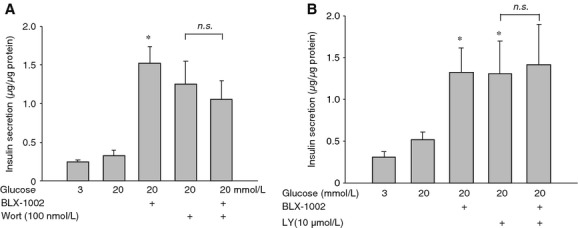
The effect of BLX‐1002 on glucose‐induced insulin secretion involves PI3K activity. Islets isolated from diabetic GK rats were pretreated with or without PI3K inhibitors wortmannin (Wort A) or LY‐294002 (Ly B) for 15 min, followed by 30 min incubation with or without BLX‐1002. Bars represent mean ± SEM. Results are derived from four experiments (*P <**0.05 vs. 3 mmol/L glucose by ANOVA).

We previously showed that BLX‐1002 potentiates GSIS in pancreatic *β*‐cells from nondiabetic obese mice, an action that requires PI3K activity (Zhang et al. 2009). In order to understand whether PI3K activity was also involved in the effect of BLX‐1002 on GSIS in the diabetic islet cells, the islets were incubated with BLX‐1002 in the presence of glucose after a brief pretreatment with the PI3K‐specific inhibitors wortmannin or LY294002 (Fig. 5). It is known that PI3K activity normally plays a crucial role in the suppression of GSIS. Conversely, suppression of PI3K activity in islet cells by wortmannin or LY294002 potentiates GSIS (Eto et al. 2002). The diabetic islets responded to PI3K inhibition with increased insulin secretion. Incubation of the islets with either wortmannin (Fig. 5A) or LY294002 (Fig. 5B) led to enhanced insulin secretion in the presence of glucose. Coincubation of the inhibitors with BLX‐1002 did not show any effect above those induced by the PI3K inhibitors.

## Discussion

We show here for the first time that the novel thiazolidinedione BLX‐1002 restores GSIS in diabetic GK rat islets normally “blind” to glucose. Basal insulin secretion (at low glucose) was not affected, whereas the drug also potentiated insulin secretion stimulated by both GLP‐1 and sulfonylurea and only in diabetic GK rat islets. All of these effects are very attractive from a therapeutic point of view: (1) the drug worked only in diabetic islets and not in nondiabetic ones, where there is no need to increase insulin output; (2) no effects occurred at nonstimulatory glucose concentrations, which may minimize the risk of hypoglycemia; (3) as loss of GSIS is a selective and early lesion in T2DM, its restoration by BLX‐1002 normalizes first phase insulin secretion needed to curb hepatic glucose production and thus prandial glycemia; (4) BLX‐1002 augmented the secretory stimulation evoked by two commonly used antidiabetic agents (GLP‐1 and sulfonylurea); thus, it may work as add‐on therapy to any of these; (5) the metabolite BLX‐1237 was inactive, making it easier to predict the pharmacological response to BLX‐1002 by pharmacokinetic and pharmacodynamics analyses. Again, this may prove clinically advantageous as it may minimize prolonged hypoglycemic effects by drug metabolites, a major problem with many sulfonylurea drugs (Hussain et al. 2014). In our previous paper (Zhang et al. 2009), we found a clear dose‐dependent stimulation of insulin secretion in the range 0.1–10 *μ*mol/L. BLX‐1002 C_max_ in rodents is in the submicromolar–micromolar range (Deben Dey, unpublished), making our in vitro findings potentially relevant for the in vivo situation. There is an apparent discrepancy between Figures 1 and 2A in the GSIS induced by BLX‐1002, in that the secretory response to 10 mmol/L glucose is much less potentiated by BLX‐1002 than 20 mmol/L glucose. It is possible that this reflects the fact that the ambient glycemia in vivo in GK rats is around 10 mmol/L and thus that this is the “normal” level of glycemia for GK islets.

We also started to address the mechanistic basis for these effects of BLX‐1002. A rise in [Ca^2+^]_i_ is an important event regulating early phase insulin secretion (Wollheim and Sharp 1981; Ashcroft and Rorsman 2012). Entirely consistent with such a role, insulin secretion potentiated by BLX‐1002 at high glucose was associated with an enhanced cytoplasmic‐free Ca^2+^ concentration ([Ca^2+^]_i_) in the *β*‐cell. Our data indicate that BLX‐1002 rapidly enhances [Ca^2+^]_i_ stimulated not only by high glucose, but also by GLP‐1 and tolbutamide. Although further work is clearly needed to pin down the precise nature of the effects exerted by BLX‐1002, it is noteworthy that we found no effects of the drug on K_ATP_ channel (or voltage‐gated Ca^2+^ channel) activity in our previous paper in normal mouse islets (Zhang et al. 2009). Our present findings also show that extracellular Ca^2+^ is required, as the effect of BLX‐1002 on GSIS vanished in the absence of extracellular Ca^2+^, whereas acetylcholine (which mobilizes Ca^2+^ from intracellular stores) remained effective. GSIS potentiated by BLX‐1002 was not augmented by specific inhibitors of PI3K, a key element in transducing insulin signaling (Virkamäki et al. 1999) and controlling vesicle trafficking and secretory function in many cell types (De Camilli et al. 1996; Rameh and Cantley 1999). In addition, an important role of the enzyme in *β*‐cell secretory function has been suggested (Eto et al. 2002). However, recent work has shown that the picture may be more complex than previously thought: It appears that the *α* and *β* catalytic subunits of type 1A PI3K have diametrically opposite effects on insulin secretion (Kolic et al. 2013). Again, further work is needed to elucidate the exact molecular mechanisms by which BLX‐1002 exerts its insulinotropic effects and we encourage interested readers to engage in this process.

In human T2DM, loss of glucose‐sensitive insulin secretion is an important pathogenic event even in the very earliest stages of disease progression (Pfeifer et al. 1981). The mechanisms underlying this functional defect remain elusive, but are also attractive targets for attempts to prevent or block *β*‐cell failure. Hence, improvement of *β*‐cell function is a major goal not only in diabetes research, but also in the clinical management of the disease. Thus, an issue pertinent to addressing the relentless decline in *β*‐cell function in T2DM (and thus the progressive course of the disease) is whether drug treatments negatively or positively impact *β*‐cell function over the long term. Although any intervention decreasing hyperglycemia may improve *β*‐cell function by reducing glucose toxicity, certain antidiabetic drugs may have direct *β*‐cell effects per se. The UKPDS clearly showed the progressive decline in glycemic control in newly diagnosed T2DM patients, irrespective of the kind of drug therapy (by drugs available at the time, Tourrel et al. 2001). More contemporary studies have even shown that sulfonylureas may accelerate loss of glycemic control, whereas PPAR‐*γ* agonists and GLP‐1‐based drugs may slow down this process (Kahn et al. 2006). Such clinical findings align well with reports in vitro, showing that sulfonylureas may induce apoptotic death of human *β*‐cells (Maedler et al. 2005), whereas PPAR‐*γ* agonists (Zeender et al. 2004; Zhang et al. 2006a) and GLP‐1 receptor activators (Holz et al. 1993; Tourrel et al. 2001) may protect *β*‐cells against such a death and render them more glucose sensitive.

In conclusion, our findings suggest that BLX‐1002 potentiates insulin secretion by different stimuli in diabetic, but not in nondiabetic, *β*‐cells in a Ca^2+^‐dependent manner involving PI3K. Although speculative, the mechanisms involved may translate into substantial clinical benefits of BLX‐1002 in the management of type 2 diabetes – which differentiates it from most currently available antidiabetic drugs – by avoidance of prolonged hypoglycemia, lack of weight gain, selectivity for diabetic *β*‐cells, restoration of early GSIS and [Ca^2+^]_i_ rise with attendant recovery of prandial glycemic control, and its suitability for combination therapy with sulfonylureas or GLP‐1‐based therapy.

## Conflicts of Interest

None declared.
